# Supplementary material for: Idiopathic epiretinal membrane area changes in eyes with good vision and the association with visual function
Source: PLoS One. 2025 Sep 2;20(9):e0331437. doi: 10.1371/journal.pone.0331437 (PMC12404372; doi:10.1371/journal.pone.0331437)
Supplement: S1 Table — (DOCX) [file pone.0331437.s002.docx]

**S1 Table. Comparison of retinal thickness changes between the two groups at baseline and final follow-up.**

|  | Baseline | | | Follow-up | | | Thickness change | | |
| --- | --- | --- | --- | --- | --- | --- | --- | --- | --- |
| Variable | Progression group | Stable group | P-value | Progression group | Stable group | P-value | Progression group | Stable group | P-value |
| Total retinal thickness |  |  |  |  |  |  |  |  |  |
| Central 1mm ring (um) | 373.0 ± 56.4 | 360.5 ± 52.9 | 0.442 | 402.3 ± 64.7* | 375.7 ± 50.6* | 0.129 | 29.3 ± 45.4 | 15.3 ± 20.7 | 0.186 |
| Inner 3mm ring (um) | 363.5 ± 27.2 | 359.1 ± 35.2 | 0.639 | 378.8 ± 38.3* | 365.5 ± 35.8* | 0.228 | 15.3 ± 26.7 | 6.4 ± 10.9 | 0.142 |
| Outer 6mm ring (um) | 296.0 ± 17.1 | 294.5 ± 25.5 | 0.818 | 298.2 ± 24.4 | 297.6 ± 28.0 | 0.938 | 2.2 ± 12.9 | 3.1 ± 7.2 | 0.776 |
| Inner retinal thickness |  |  |  |  |  |  |  |  |  |
| Central 1mm ring (um) | 115.3 ± 42.0 | 107.9 ± 32.2 | 0.504 | 137.1 ± 50.7* | 116.5 ± 34.8* | 0.166 | 21.8 ± 37.0 | 8.7 ± 15.1 | 0.121 |
| Inner 3mm ring (um) | 152.8 ± 19.6 | 154.5 ± 23.2 | 0.791 | 160.6 ± 25.2* | 157.0 ± 23.5 | 0.625 | 7.8 ± 18.0 | 2.5 ± 7.8 | 0.207 |
| Outer 6mm ring (um) | 124.5 ± 11.7 | 128.3 ± 17.1 | 0.382 | 125.4 ± 17.4 | 129.4 ± 21.1 | 0.486 | 0.9 ± 10.2 | 1.1 ± 8.9 | 0.945 |
| Outer retinal thickness |  |  |  |  |  |  |  |  |  |
| Central 1mm ring (um) | 257.7 ± 22.4 | 252.6 ± 28.9 | 0.509 | 265.5 ± 20.3* | 259.2 ± 26.4* | 0.408 | 7.8 ± 18.2 | 6.6 ± 13.8 | 0.863 |
| Inner 3mm ring (um) | 210.7 ± 12.6 | 204.6 ± 15.6 | 0.154 | 218.2 ± 16.0* | 208.4 ± 16.0* | 0.097 | 7.6 ± 9.3 | 3.8 ± 4.6 | 0.092 |
| Outer 6mm ring (um) | 171.5 ± 7.5 | 166.2 ± 10.5 | 0.056 | 172.8 ± 8.8 | 168.1 ± 11.0 | 0.124 | 1.3 ± 5.4 | 2.0 ± 6.1 | 0.691 |

Comparisons between groups were performed using independent t-tests or Mann–Whitney U tests.

Comparisons between two dependent samples were performed using the paired sample t-test or Wilcoxon signed-rank test.

*Statistically significant difference between baseline and final follow-up in each group (P<0.05)
