# Supplementary material for: Idiopathic epiretinal membrane area changes in eyes with good vision and the association with visual function
Source: PLoS One. 2025 Sep 2;20(9):e0331437. doi: 10.1371/journal.pone.0331437 (PMC12404372; doi:10.1371/journal.pone.0331437)
Supplement: S2 Table — (DOCX) [file pone.0331437.s003.docx]

**S2 Table. Correlation between ERM area changes and retinal thickness changes**

|  | Stable group |  | Progression Group |  |
| --- | --- | --- | --- | --- |
| Variable | Pearson’s coefficient | P-value^#^ | Pearson’s coefficient | P-value |
| Total area reduction ratio vs. |  |  |  |  |
| Central 1mm ring^*^ | -0.164 | 0.455 | 0.033 | 0.880 |
| Inner 3mm ring | -0.321 | 0.135 | -0.110 | 0.618 |
| Outer 6mm ring | -0.169 | 0.440 | -0.170 | 0.437 |
| Superior quadrant area reduction ratio vs. |  |  |  |  |
| Central 1mm ring (um) | -0.431 | **0.040** | 0.312 | 0.148 |
| Inner 3mm ring (um) | -0.575 | **0.004** | 0.269 | 0.215 |
| Outer 6mm ring (um) | -0.439 | **0.036** | 0.014 | 0.949 |
| Inferior quadrant area reduction ratio vs. |  |  |  |  |
| Central 1mm ring (um) | -0.032 | 0.883 | 0.010 | 0.965 |
| Inner 3mm ring (um) | 0.219 | 0.316 | -0.137 | 0.533 |
| Outer 6mm ring (um) | 0.252 | 0.246 | -0.384 | 0.070 |
| Nasal quadrant area reduction ratio vs. |  |  |  |  |
| Central 1mm ring (um) | 0.016 | 0.941 | 0.016 | 0.944 |
| Inner 3mm ring (um) | -0.072 | 0.745 | -0.138 | 0.530 |
| Outer 6mm ring (um) | 0.081 | 0.713 | -0.001 | 0.996 |
| Temporal quadrant area reduction ratio vs. |  |  |  |  |
| Central 1mm ring (um) | -0.063 | 0.776 | -0.541 | **0.008** |
| Inner 3mm ring (um) | 0.028 | 0.900 | -0.463 | **0.026** |
| Outer 6mm ring (um) | 0.147 | 0.504 | -0.005 | 0.983 |

^*^Total retinal thickness

^#^Statistically significant difference between baseline and final follow-up (P<0.05)
