# Supplementary material for: Idiopathic epiretinal membrane area changes in eyes with good vision and the association with visual function
Source: PLoS One. 2025 Sep 2;20(9):e0331437. doi: 10.1371/journal.pone.0331437 (PMC12404372; doi:10.1371/journal.pone.0331437)
Supplement: S3 Table — (DOCX) [file pone.0331437.s004.docx]

**S3 Table. The values of sensitivity, specificity, and AUROC for each ratio.**

| Ratio (%) | Sensitivity | 1 - Specificity | Specificity |
| --- | --- | --- | --- |
| -5.130 | 1.000 | 1.000 | 0.000 |
| -2.245 | 1.000 | 0.957 | 0.043 |
| -0.180 | 1.000 | 0.913 | 0.087 |
| 0.838 | 1.000 | 0.870 | 0.130 |
| 1.711 | 1.000 | 0.826 | 0.174 |
| 2.078 | 1.000 | 0.783 | 0.217 |
| 2.783 | 1.000 | 0.739 | 0.261 |
| 3.281 | 1.000 | 0.696 | 0.304 |
| 3.443 | 1.000 | 0.652 | 0.348 |
| 3.525 | 1.000 | 0.609 | 0.391 |
| 3.611 | 1.000 | 0.565 | 0.435 |
| 3.787 | 1.000 | 0.522 | 0.478 |
| 4.049 | 1.000 | 0.478 | 0.522 |
| 4.219 | 1.000 | 0.435 | 0.565 |
| 4.740 | 1.000 | 0.391 | 0.609 |
| 5.454 | 0.955 | 0.391 | 0.609 |
| 5.886 | 0.909 | 0.391 | 0.609 |
| 6.402 | 0.909 | 0.348 | 0.652 |
| 6.739 | 0.909 | 0.304 | 0.696 |
| 6.838 | 0.864 | 0.304 | 0.696 |
| 7.002 | 0.818 | 0.304 | 0.696 |
| 7.310 | 0.818 | 0.261 | 0.739 |
| 7.570 | 0.818 | 0.217 | 0.783 |
| 7.657 | 0.818 | 0.174 | 0.826 |
| 7.810 | 0.773 | 0.174 | 0.826 |
| 8.031 | 0.727 | 0.174 | 0.826 |
| 8.485 | 0.682 | 0.174 | 0.826 |
| 8.962 | 0.682 | 0.130 | 0.870 |
| 9.371 | 0.682 | 0.087 | 0.913 |
| 9.855 | 0.682 | 0.043 | 0.957 |
| 10.190 | 0.636 | 0.043 | 0.957 |
| 10.951 | 0.636 | 0.000 | 1.000 |
| 11.752 | 0.591 | 0.000 | 1.000 |
| 12.472 | 0.545 | 0.000 | 1.000 |
| 13.520 | 0.500 | 0.000 | 1.000 |
| 14.176 | 0.455 | 0.000 | 1.000 |
| 14.717 | 0.409 | 0.000 | 1.000 |
| 15.141 | 0.364 | 0.000 | 1.000 |
| 16.354 | 0.318 | 0.000 | 1.000 |
| 17.795 | 0.273 | 0.000 | 1.000 |
| 18.143 | 0.227 | 0.000 | 1.000 |
| 19.742 | 0.182 | 0.000 | 1.000 |
| 22.332 | 0.136 | 0.000 | 1.000 |
| 24.558 | 0.091 | 0.000 | 1.000 |
| 26.511 | 0.045 | 0.000 | 1.000 |
| 28.297 | 0.000 | 0.000 | 1.000 |
